# Primary rhegmatogenous retinal detachment with inferior retinal breaks postoperative prone positioning results: 1 day versus 7 days

**DOI:** 10.1186/s40942-017-0100-0

**Published:** 2017-12-04

**Authors:** Radwan Ajlan, Jordan Isenberg, Ghassan Cordahi, Renaud Duval, Sébastien Olivier, Flavio Rezende

**Affiliations:** Department of Ophthalmology, Hôpital Maisonneuve-Rosemont, Université de Montréal, 5415 boul. de l’Assomption, Montreal, QC H1T 2M4 Canada

**Keywords:** Inferior retinal detachment, Head positioning, Inferior retinal break

## Abstract

**Background:**

To compare the primary anatomical outcome of pars plana vitrectomy, 360° peripheral endolaser, and 15% octafluoropropane C_3_F_8_ gas tamponade in patients with uncomplicated rhegmatogenous detachment and inferior retinal breaks, after 24-h postoperative prone positioning to similar patients with 1 week postoperative prone positioning.

**Methods:**

Records of 5500 patients who underwent pars plana vitrectomy between 2008 and 2015 were retrieved. Collected data included age, gender, number of retinal quadrants with retinal breaks, number of retinal breaks, macula status on presentation (attached or detached), phakic status (phakic, pseudophakic, or aphakic), and primary anatomical outcome (at 1 and 3 months post-operative).

**Results:**

270 patients met the study inclusion criteria (78 females, and 192 males). In the 24-h positioning arm (183 patients), the overall anatomical success rate was 96.2% at 1 month and 83.6% at 3 months. In the 1-week positioning group (87 patients), the overall anatomical success rate was 93% at 1 month and 79% at 3 months. Both positioning groups did not show statistical difference in outcome at 1 month (p-value = 0.7) or at 3 months (p-value = 0.39). Logistic regression analysis found that the number of retinal breaks correlates with the postoperative anatomical success at 3 months (odd ratio 0.8, p-value = 0.016).

**Conclusion:**

This short term retrospective study demonstrated that patients with uncomplicated rhegmatogenous retinal detachment due to inferior retinal breaks, who underwent pars plana vitrectomy, 360° endolaser, 15% C_3_F_8_ gas, and limited (24-h) prone positioning did not show statistical difference in the anatomical outcome (at 1, and 3 months) when compared with 1 week postoperative positioning. Larger prospective studies are warranted to further elucidate positioning role.

## Background

Retinal detachment is a vision threatening disease that has been reported to range between five and 12 cases per 100,000 population per year [1–3], with a lifetime risk of 0.6% (up to 60 years of age) [4, 5]. Although 6% of the general population are thought to have retinal breaks, most of these are asymptomatic benign atrophic holes, which are without accompanying pathology, and do not lead to retinal detachment [4].

In the past, pars plana vitrectomy (PPV) was used as the primary surgical intervention for only complicated retinal detachments. Nonetheless, favorable reattachment rates with low intraoperative complication rates have made PPV an increasingly popular primary option for rhegmatogenous retinal detachment (RRD) repair [6]. Technological advancements allow the surgeon to visualize all retinal breaks, as well as the removal of opacities. On the other hand, Afrashi et al. [7] reported iatrogenic retinal breaks in more than 7% of patients, and proliferative vitreoretinopathy (PVR) in more than 9% after PPV.

Inferior breaks have also been reported to be associated with surgical failure following PPV for RRD repair [8–10]. The scleral buckling versus primary vitrectomy in rhegmatogenous retinal detachment study (SPR group) showed that inferior detachment with breaks below the 4 and 8 o’clock positions was a significant risk factor in pseudophakic/aphakic subtrial [11].

There is little agreement on the optimal postoperative head positioning duration in patients with RRD due to inferior retinal break after PPV and intraocular long-acting gas infusion. Some surgeons prone position these patients for 1 day, while others recommend positioning for 7, or 14 days.

## Purpose

To compare 24-h and 1 week postoperative prone positioning anatomical outcome after PPV, 360° peripheral endolaser, and 15% C_3_F_8_ gas tamponade in patients with uncomplicated RRD and inferior retinal breaks.

## Methods

This is a retrospective cohort study, with two main comparative arms based on postoperative prone head positioning duration (1 day vs. 7 days). Postoperative positioning duration was assigned based on surgeons’ practice pattern, 1 surgeon only recommends positioning for 1 day, while the other 3 surgeons recommend positioning for 7 days for retinal detachment with inferior breaks. Primary outcome for this study is anatomical outcome at 1 month postoperative visit, while the secondary outcome is anatomical outcome at 3 months postoperative visit.

**Fig. 1:**
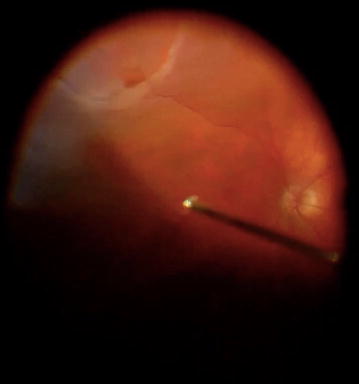
Intraoperative fundus view of right eye, showing inferior rhegmatogenous retinal detachment secondary to inferior retinal break

Records of patients who underwent PPV at the University of Montreal affiliated ophthalmology department at Maisonneuve-Rosemont Hospital (HMR) in Montreal, Canada between 2008 and 2015 were reviewed. Inclusion criteria include primary RRD secondary to inferior retinal breaks (Fig. 1) that was treated with primary PPV, 360° peripheral endolaser, and 15% C_3_F_8_ gas tamponade. Exclusion criteria include history of previous retinal detachment repair, scleral buckling, any degree of proliferative vitreoretinopathy (PVR), exudative retinal detachment, retinoschisis related detachment, noncompliance with postoperative treatment (as per medical records documentation), pregnant women, or pediatric population.

Data collection included: age, gender, location of retinal breaks, number of retinal breaks, macula attachment status on presentation, intraocular lens status (phakic, pseudophakic, aphakic), anatomical outcome (at 1 month, and 3 months), and postoperative prone head positioning duration (1 day vs. 7 days). Ethics approval was granted by the ethics review board at HMR for research and publication, no consent form was needed. None of the authors have any competing interests in the manuscript. Statistical analysis was conducted using Stata Statistics/Data Analysis software v.14. All results generated from this study are included in this published article (study data can be accessed on obtaining the host institute ethics board approval).

**Fig. 2:**
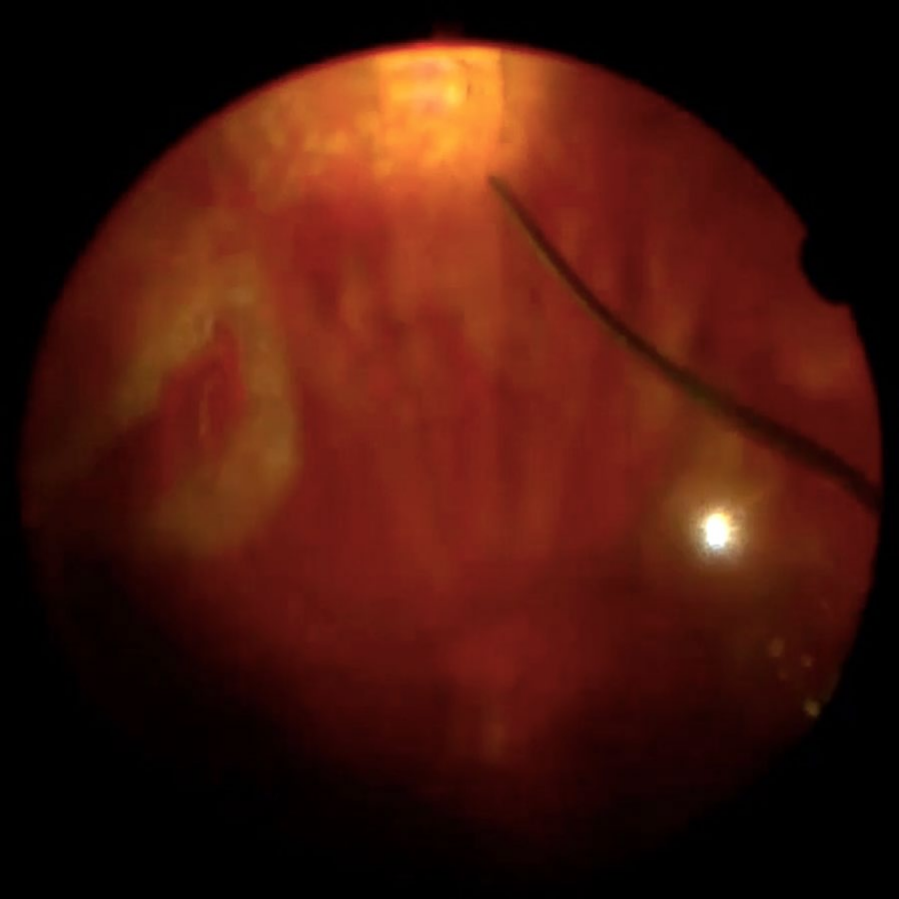
Intraoperative fundus view of right eye, showing laser treated inferior retinal break, and the beginning of the 360° laser treatment with 3–4 lines of moderate intensity laser burns

Surgical technique involves retrobulbar block using either xylocaine 2% (lidocaine hydrochloride) solely, or mixed with Marcaine 0.5% (bupivacaine hydrochloride) in a 50:50 mix. A 23-gauge or 25-gauge vitrectomy was conducted depending on surgical year (25-gauge starting 2013). All patients had scleral depression assisted peripheral vitreous shave releasing traction and removing any retinal tear flap (Fig. 2), with three to four lines of medium intensity endolaser treatment 360° at the retinal far periphery (532 nm wavelength green laser, CONSTELLATION^®^ Vision System, Alcon, Canada) (Fig. 2). After fluid-air exchange, Perfluoropropane (octafluoropropane C_3_F_8_) gas in non-expansile 15% concentration was infused intraoculary. Patients’ head positioning began after termination of surgery before exiting the surgical suite.

## Results

Initially 5500 patients were identified, with 270 meeting the inclusion criteria (78 females, and 192 males). Mean age for patients was 66 ± 13 years for the 1 day group and 65 ± 13 years for the 7 days group (p = 0.3), with females compromising 31.1 and 26.4% of the 1 day and the 7 days positioning groups respectively (p = 0.54). In addition, no statistical difference between groups for intraocular lens status (31.7 and 24.1% were phakic in the 1 day group, and the 7 days groups respectively, p = 0.2), or macular status on presentation was detected (52 and 42.5% had the macula attached in the 1 day and 7 days group respectively, p = 0.15). Number of retinal breaks was found to be statistically different between groups (mean number of breaks was 1.9 ± 0.8 in the 1 day group and 2.6 ± 1.7 in the 7 days group, p = 0.002), meanwhile the number of quadrants detached was found to be marginally significant between groups (mean number of quadrants detached was 1.9 ± 0.8 and 2.1 ± 0.9 for the 1 day group, and the 7 days group respectively, p = 0.037) (Table 1).

**Table 1:** Demographic distribution of study population

	1 day positioning group (N* = 183)	7 days positioning group (N* = 87)	p value
Age (mean)	66 ± 13 years	65 ± 13 years	0.3
Gender, females (N*, %)	55 (30.1%)	23 (26.4%)	0.54
Lens status, phakic (N*, %)	58 (31.7%)	21 (24.1%)	0.2
Number of retinal breaks (mean ± SD*)	1.9 ± 0.8	2.6 ± 1.7	0.002
Number of quadrants detached (mean ± SD*)	1.9 ± 0.8	2.1 ± 0.9	0.037
Macula attached on presentation (N*, %)	95 (52%)	37 (42.5%)	0.15

N*, number; SD, standard deviation

### One day positioning group results

**Fig. 3:**
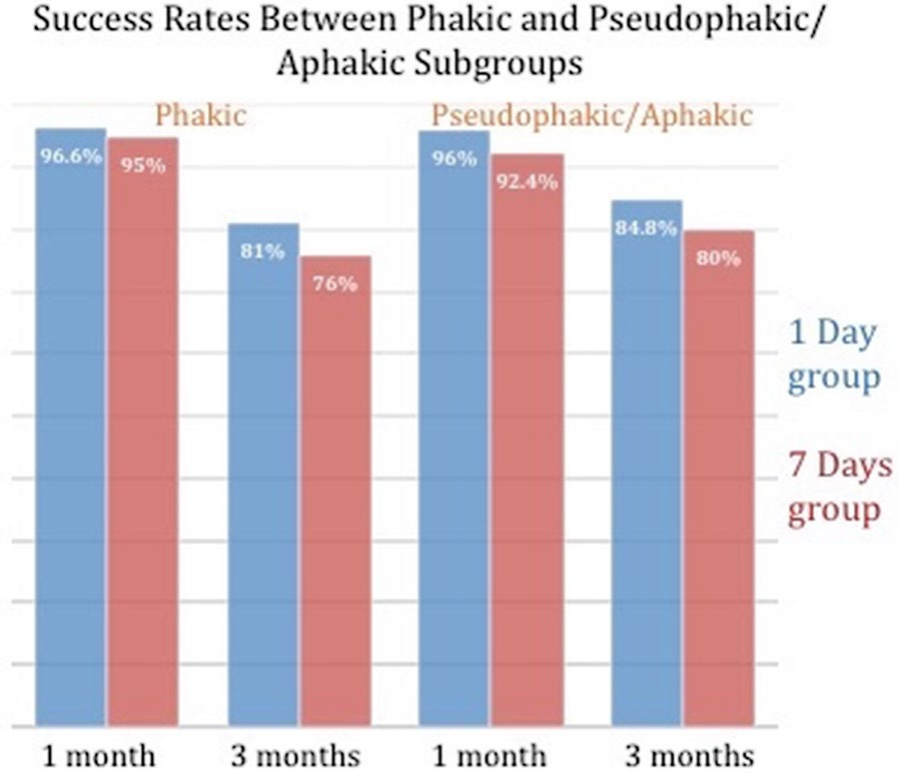
Graphic figure demonstrating success rates between phakic and pseudophakic/aphakic subgroups at 1 month and at 3 months post PPV

In the 24-h positioning arm (183 patients), overall primary anatomical success rate was 96.2% at 1 month, and 83.6% at 3 months. Primary anatomical success in phakic subgroup (58 eyes) was 96.6% at 1 month, and 81% at 3 months post-operative. In the pseudophakic/aphakic subgroup (125 eyes), primary anatomical success was 96%, and 84.8% at 1 month and at 3 months post-operative visits respectively (Fig. 3).

### Seven days positioning group results

In the 1 week positioning group (87 patients), the overall primary anatomical success rate was 93% at 1 month, and 79% at 3 months. Primary anatomical success in phakic subgroup (21 eyes) was 95% at 1 month, and 76% at 3 months post-operative. In the pseudophakic/aphakic subgroup (66 eyes), primary anatomical success was 92.4%, and 80% at 1 month, and at 3 months post-operative visits respectively (Fig. 3).

**Fig. 4:**
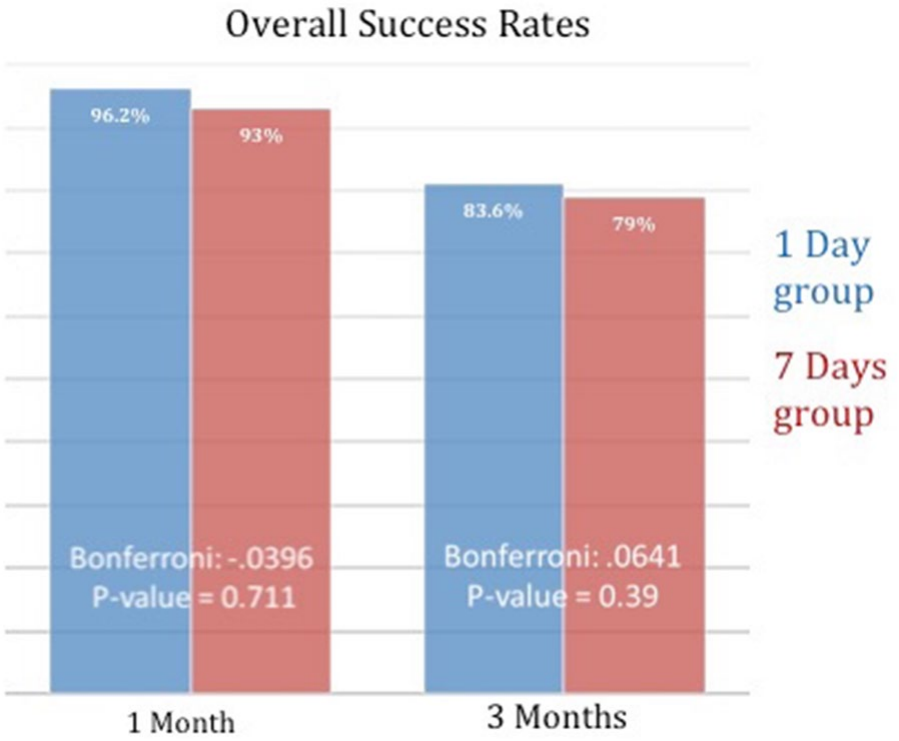
Overall success rate after 1 month was 96.2% for the 1 day positioning group, and 93% for the 7 days positioning group. At the 3 months point, anatomical success rate was 83.6% for the 1 day positioning group, and 79% for the 7 days positioning group. Statistical analysis did not find significant differences between both groups either at 1 month or at 3 months points

No statistical difference in primary anatomical outcome was detected between groups at 1 month (p value = 0.7), or at 3 months (p value = 0.39) (Fig. 4).

When controlling for age, gender, macula attachment on presentation, lens status, number of quadrants detached, number of retinal breaks, and postoperative positioning duration, logistic regression analysis found that only number of retinal breaks statistically correlated with postoperative anatomical success at 2 months (odd ratio 0.8, p = 0.016) (Table 2).

**Table 2:** Logistic regression analysis, when controlling for gender, macula attachment, lens status, number of quadrant detached, and postoperative positioning group, the number of retinal breaks is found to statistically correlate with the postoperative anatomical success at 3 months (odds ratio 0.78, p value = 0.017)

Three months outcome	Odds ratio	SE	p value	95% CI	
Positioning duration	2.664441	2.189294	0.233	0.53232514	13.33563
Number of breaks	0.7807092	0.0801036	0.016	0.6384882	0.9546095
Number of quadrant detached	1.476871	0.3278644	0.079	0.9558208	2.281964
IOL status	0.6271382	0.2264768	0.196	0.3090089	1.272786
Macula status on presentation	1.086535	0.3995302	0.821	0.5285033	2.233775
Gender	1.360931	0.5239716	0.423	0.6399044	2.894389
Age	1.004502	0.012828	0.725	0.9796715	1.029962

## Discussion

Recent advances in the development and use of intravitreally injected substitutes has played an important role in facilitating retinal reattachment surgery [6]. Intraocular gases help prevent intravitreal fluid escape through retinal breaks to the subretinal space by closure of retinal breaks using high surface tension interface, mechanical buoyance keeping retina attached, and decrease intraocular fluid current over retinal breaks. Nevertheless, when causative breaks are located inferiorly, the facedown position, and long-acting gas have been recommended [12–16]. However, patients who receive gas tamponade must be monitored closely as gases have been associated with natural lens opacification and high intraocular pressure.

Maintenance of the position after surgery when a gas tamponade is used is considered essential for achieving success in retinal detachment (RD) surgery. Nonetheless, some patients are unable to position themselves appropriately after surgery owing to medical, physical, or social conditions. To avoid this problem, different authors have used a buckle combined with PPV for the management of these breaks [17–19]. However, a recent multicenter study in Europe included over 7000 patients, found that when a PPV is done in uncomplicated RD, a supplemental buckle was not found to be beneficial [20]. Others have used silicone oil as a tamponade [21]. Some reports proposed the use of air alone as a tamponade for the management of inferior breaks in pseudophakic RD without facedown positioning in 40 patients with primary success rate of 90% [22], or only air with 24-h prone position in a different series of 15 patients with primary success rate of 93.3% [23]. A recent series included 147 patients proposed the use of vitrectomy only to treat retinal detachment secondary to inferior retinal breaks, including fluid–air exchange, treating only retinal breaks with transscleral laser or endolaser, and no face-down positioning, initial reattachment rate was reported to be 94.5% [24].

Since PPV primary success rate is reported to be less than 90% in several major epidemiological studies [20, 25, 26], the decision of including a control group arm that has all reported success factors (including PPV, no PVR, C_3_F_8_ gas, prophylactic 360° laser) was stressed in an attempt to have only one variable between groups, which was the duration of postoperative head positioning. The 1 month end point was designed to assess anatomical outcome while the C_3_F_8_ gas bubble is still in the eye, has decreased by approximately 50%, no longer covering the inferior breaks, and after laser treated areas have formed scarred adhesion. The second end point of 3 months was chosen to allow intraocular C_3_F_8_ gas to completely resorb [27], as a result, there would be no gas bubble to conceal untreated superior pathology (e.g. superior retinal break, or PVR).

Argon laser photocoagulation is found to enhance retina adhesiveness within 24 h to 128% of normal levels in rabbit eyes, while cryoretinopexy reduced the retinal adhesiveness during the first week, but afterwards generated as much adhesiveness as photocoagulation. Beyond 6 months, the adhesive force for cryoretinopexy was (214%), and for laser photocoagulation was (220%) [28].

Yoon et al. examined how quickly laser photocoagulation produces a bond between retina and pigment epithelium. After photocoagulation of intact retina, the adhesive force was reduced 50% at 8 h but increased beyond normal (to approximately 140%) by 24 h and remained twice normal between 3 days and 3 weeks. Photocoagulation of retina that had just settled after experimental detachment produced similar results, except that the maximum strength of adhesion (reached at 2 weeks) was three times normal. In contrast, retina that reattached spontaneously, without photocoagulation, showed only 10% of normal adhesiveness at 24 h, and still was only 75% of normal after 4 weeks. These data suggest that laser photocoagulation may be acutely beneficial in the prevention or management of retinal tears and detachments [29]. which served as part of the rational for the 1 day positioning duration by one surgeon. On the other hand, excessive laser treatment can lead to progressive localized retinal atrophy and development of future retinal breaks at the margin of the laser scar.

Conversely, the application of prophylactic 360 laser retinopexy may be an effective method of reducing the incidence of retinal redetachment in the same eye. Laidlaw et al. [30] found a significant reduction in the incidence of retinal redetachment after the removal of silicone oil tamponade in patients undergoing laser retinopexy. Intraoperative 360° laser retinopexy following phaco-vitrectomy resulted in a significant reduction in the rate of postoperative retinal detachment in macular hole cases, or in epiretinal membrane peel surgeries combined with phacoemulsification [31, 32]. That was the rational for including 360° laser retinopexy in the inclusion criteria, and the reason it was conducted by participating surgeons retrospectively.

On the other hand, it has been proven that subbasal corneal nerve plexus density decreases after 360° laser retinopexy, and is accompanied by epithelium thinning and decreased corneal sensation. As a result, it is recommended that heavy confluent retinal laser treatment should be avoided when possible, and corneal sensitivity be assessed postoperatively to determine whether significant anesthesia has occurred. In such instances, prophylactic measures may be warranted against the development of neurotrophic ulcers [33].

This study is a retrospective study, which creates a challenge in having balanced groups. To avoid any selection bias, all patients meeting the inclusion criteria were included, that resulted in the number of included patients to be more in the 1 day positioning group compared to the 7 days positioning group (183, and 87 patients respectively). However, on sample demographic analysis no statistical difference was detected comparing age, gender, lens status, or macular attachment on presentation. There was a statistical difference in the number of retinal breaks (1.9 ± 0.8, and 2.6 ± 1.7, p value = 0.002), and a marginal difference in number of quadrants detached (1.9 ± 0.8, and 2.1 ± 0.9, p value = 0.037) between groups (1 day, and 7 days positioning groups respectively).

## Conclusion

This short term retrospective study demonstrated that patients with uncomplicated rhegmatogenous retinal detachment due to inferior retinal breaks, who underwent pars plana vitrectomy, 360° endolaser, 15% C_3_F_8_ gas, and limited (24-h) prone positioning did not show statistical difference in the anatomical outcome (at 1, and 3 months) when compared with 1 week postoperative positioning. Larger prospective studies are still warranted to further elucidate postoperative positioning role in similar cohort of patients.
